# Active prospective national surveillance for congenital and neonatal varicella in Australia shows potential prevention opportunities

**DOI:** 10.1016/j.jvacx.2023.100278

**Published:** 2023-02-18

**Authors:** Jacina Walker, Suzy Teutsch, Anne Morris, Guy D. Eslick, Mahmudul Hassan Al Imam, Arifuzzaman Khan, Robert Booy, Elizabeth J Elliott, Gulam Khandaker

**Affiliations:** a Central Queensland Public Health Unit, Central Queensland Hospital and Health Service, Rockhampton, Queensland, Australia; b The University of Sydney, Faculty of Medicine and Health, Discipline of Child and Adolescent Health, Sydney, New South Wales, Australia; c Australian Paediatric Surveillance Unit, Kids Research, Sydney Children’s Hospitals Network (Westmead), Sydney, New South Wales, Australia; d The Sydney Children's Hospitals Network, Westmead, Sydney, New South Wales, Australia; e School Health, Medical and Applied Sciences, Central Queensland University, Rockhampton, Queensland, Australia; f School of Public Health, The University of Queensland, Brisbane, Australia; g National Centre for Immunisation Research and Surveillance, Sydney, New South Wales, Australia

**Keywords:** Epidemiology, Congenital varicella, Neonatal varicella infection, Vaccination, Australia, APSU, Australian Paediatric Surveillance Unit, CRF, Case Report Form, CVS, Congenital Varicella Syndrome, NVI, Neonatal Varicella Infection, CIs, Confidence Intervals, NIP, National Immunisation Program, VZV, Varicella-Zoster Virus, ZIG, Zoster immune globulin

## Abstract

To compare the incidence and outcomes of congenital and neonatal varicella in Australia in the pre-vaccination (1995–1997) and post-vaccination era (after 2005 to November 2020), active prospective national surveillance for congenital varicella syndrome (CVS) and neonatal varicella infection (NVI) was conducted through the Australian Paediatric Surveillance Unit (APSU). Compared with 1995–1997, there was a 91.5% reduction in the incidence of CVS and a 91.3% reduction in the incidence of NVI in 2009–2020. However, almost half of the mothers in 2009–2020 were born overseas and came from countries without a vaccination program. Although there has been a substantial and sustained decrease in the reported incidence of CVS and NVI in Australia since 2006, congenital and neonatal varicella infections persist. Thus, there is an opportunity for targeted screening of varicella in young migrant, asylum seeker and refugee women at risk of varicella infection and prioritisation for vaccination to prevent CVS and NVI.

## Highlights

• The incidence of congenital and neonatal varicella infection in Australia has decreased substantially since the introduction of a universal varicella vaccination program in 2005.
• There is a high risk of imported varicella infection among immigrant populations from countries without similar varicella vaccination programs.
• Rates of maternal varicella infection and subsequent congenital and neonatal varicella infection could be reduced if newly arrived immigrants were more comprehensively screened for vaccine-preventable infections, including varicella as part of their health check when entering Australia.

## 1 Introduction

Maternal varicella-zoster virus (VZV) infection within the first 20 weeks of pregnancy can cause serious complications for the mother and baby and may be associated with congenital varicella syndrome (CVS) [1], [2], [3]. CVS can result in spontaneous abortion, stillbirth, premature delivery, structural eye damage, neurological abnormalities and characteristic skin lesions including cicatricial lesions with a dermatomal distribution [4]. CVS may also present as herpes zoster in the first 12 months of life. On the other hand, neonatal varicella infection (NVI) may result from either intrauterine or early postnatal VZV infection, and maternal varicella infection within five days before and two days after delivery is associated with disseminated or more severe neonatal disease with up to 20% mortality [5], [6].

Data on the epidemiology of CVS and neonatal varicella infection in Australia have previously been collected in two studies conducted by the Australian Paediatric Surveillance Unit (APSU). In the first study (1995 to 1997), six cases of CVS were identified, with an annual incidence of 0.8 per 100,000 live births per year for the whole study period and an average of 2.3 cases per year [7]. That study also identified 44 cases of NVI with an annual incidence of 5.8 per 100,000 live births per year and an average of 14.7 cases per year.

Public funding in Australia for a live attenuated VZV vaccine was made available by the National Immunisation Program (NIP) in November 2005 [8]. The second APSU study (2006 to 2009) commenced following the recommendation that the VZV vaccine should be given to all infants at 18 months of age and as a single catch-up dose to children aged 10–13 years. The study identified two cases of CVS with an annual incidence of 0.19 per 100,000 live births, and 16 cases of NVI with an annual incidence of 2.05 per 100,000 live births [9]. There was a reduction in the annual incidence of both CVS and NVI during 2006–2009 compared with 1995–1997 [7], however, the reduction in CVS incidence was not statistically significant.

The aim of the current study was to compare the incidence, characteristics, and outcomes of congenital and neonatal varicella in Australia between December 2009 and November 2020, with similarly collected data from June 2006 to November 2009 and the pre-vaccination era, 1995–1997.

## 2 Materials and methods

### 2.1 Surveillance timeframe and methodology

Active prospective national surveillance for CVS and NVI was conducted between 1 December 2009 and 30 November 2020 using APSU surveillance methods as previously described [7], [9]. A monthly report card listing rare conditions of interest was sent by email (95%) or post to an average of 1440 paediatricians and other child health specialists who were registered with the APSU. Clinicians were asked to report whether, or not, they had seen an infant fulfilling diagnostic criteria for CVS or NVI in the previous month. If a case was reported, clinicians were asked to complete a de-identified case report form (CRF) that included detailed information about demographics, varicella diagnosis, clinical characteristics, treatment and short-term outcomes. Data from the CRF data were entered directly online by clinicians into the secure REDCap electronic data capture system [10], [11], hosted by The University of Sydney or manually into a paper form and subsequently entered into REDCap by APSU staff. All CRFs were reviewed by study investigators with clinical expertise in varicella to confirm that case criteria were met. All data were exported to Microsoft-Excel for analysis.

### 2.2 Statistical analysis

Frequencies were calculated separately for CVS and NVI, and annual incidence rates and 95% confidence intervals (CIs) were calculated using a standard formula and expressed as per 100,00 live births per year. Live birth numbers were obtained from the Australian Institute of Health and Welfare [12] and the Australian Bureau of Statistics [13]. Descriptive statistics and a generalised linear model of Poisson distribution were computed to assess the trend of incidence rates for three APSU surveillance periods (1995–1997, 2006–2009 and 2009–2020) using RStudio, version 4.1.0 (Boston, MA, USA). An alpha level of significance of p < 0.05 was considered statistically significant.

### 2.3 Case definitions

Case definitions of CVS and NVI were as previously described [7], [9]. CVS was defined as any stillbirth, newborn infant, or child up to the age of 2 years with definite or suspected CVS, with or without birth defects, who fulfilled at least one of the following criteria:

i) Cicatricial skin lesions in a dermatomal distribution and/or pox-like skin scars and/or limb hypoplasia.
ii) Spontaneous abortion, termination, stillbirth or early death following varicella infection during pregnancy, and varicella confirmed by serology or detection of VZV or history of maternal varicella or contact with varicella during pregnancy.
iii) Development of herpes zoster in the first year of life.

NVI was defined as neonatal varicella infection in any infant in the first month of life, based on history (e.g. timing of maternal infection), clinical findings (e.g. pox-like rash and fever) and/or laboratory findings (e.g. VZV detection by culture or PCR, or IgM positive serology), but without features of CVS.

### 2.4 Ethics approval

Ethics approval for this study was obtained from the Sydney Children’s Hospitals Network Human Research Ethics Committee (HREC) (#2020/ETH03310).

## 3 Results

### 3.1 Representativeness of reporting clinicians and response rate

Between 1 December 2009 and 30 November 2020, the monthly APSU report card was sent to an average of 1440 clinicians, with 92.3% of report cards returned. Clinicians worked in all Australian states and territories, and in urban, regional and remote areas.

### 3.2 Congenital varicella syndrome

During the surveillance period (1 December 2009–30 November 2020), there were five notifications of CVS reported to the APSU with three case report forms completed and returned. Of these three reports, two children reported in 2017 and 2020, were confirmed as cases and one was classified as an error (outside the case definition).

The demographic and clinical characteristics of the two confirmed CVS cases have been previously described [14], [15]. One of the mothers was born overseas in a country without a universal VZV vaccination program (Malaysia) and the other mother was an Aboriginal Australian.

**Fig. 1:**
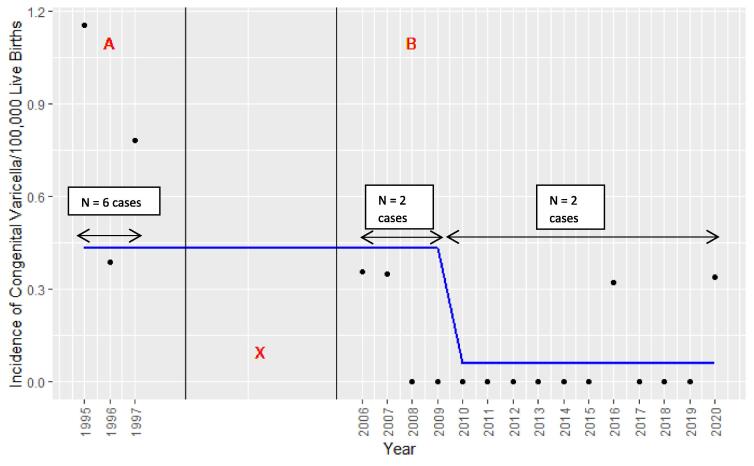
Graph showing annual incidence rates and trend line of Congenital Varicella Syndrome (CVS) obtained via Australian Paediatric Surveillance Unit (APSU) surveillance in Australia between 1995 and 2020; p = 0.146. A: Varicella vaccines were not available before 1999 [4]; B: Varicella vaccination has been incorporated in the National Immunisation Program since November 2005 [8]; X: APSU surveillance was not conducted between 1997 and 2005 so data were therefore not available.

The overall incidence estimate of CVS during the period December 2009-November 2020 was 0.07 per 100,000 live births (95% confidence interval (CI): 0.01–0.23), compared with the overall incidence estimates previously observed by us during 2006–2009 (0.19 per 100,000 live births, 95% CI: 0.0–0.7) [9] and in the pre-vaccination era 1995–1997 (0.8 per 100,000 live births, 95% CI: 0.3–1.8) [7], although this was not statistically significant (p = 0.15) (Fig. 1). Trend analysis showed there was a 91.5% decrease in the incidence of CVS during the study period compared with 1995–1997, with the number of cases declining from six in the first three years of surveillance (1995–1997) to two in the latter eleven years (late 2009–2020) (Fig. 1).

### 3.2 Neonatal varicella infection

During the surveillance period, the APSU received 20 notifications of NVI, 15 of which had a CRF completed and returned. All 15 notifications with clinical data provided were confirmed as cases. Characteristics of infants are presented in Table 1. All infants with NVI were Australian-born. Country of birth data were only available for seven of 15 mothers, of whom six were born overseas in countries without universal VZV vaccination programs, including China, Ethiopia, India and Zimbabwe.

**Table 1:** Characteristics of infants with neonatal varicella infection (NVI) in Australia, December 2009- November 2020.

***Characteristics***	**Descriptive values**
*Female Sex (%, n/N)*	50% (n = 7/14)
*Onset of illness (age in days), median*	27
*Birth weight (in grams), mean (range)*	3135; (2580–3690)
*Gestational age at birth (in weeks), mean (range)*	39; (37–41)
*Duration of illness (in days), mean (range)*	5; (1–10)
*Hospitalised (%, n/N)*	93% (n = 13/14)
*ICU admission (%, n/N)*	8% (n = 1/13)
*Received antiviral treatment (%, n/N)*	93% (n = 13/14)
*Received Zoster immune Globulin (%, n/N)*	15% (n = 2/13)
*History of varicella exposure (%, n/N)*	86% (n = 12/14, all postnatal)

**Fig. 2:**
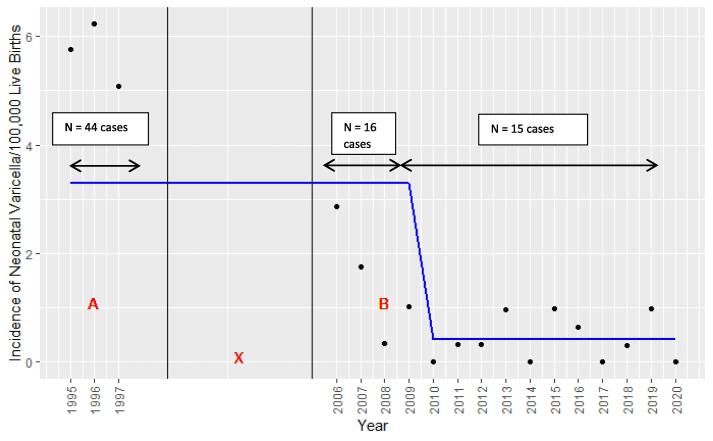
Graph showing annual incidence rates and trend line of Neonatal Varicella Infection (NVI) obtained via Australian Paediatric Surveillance Unit (APSU) surveillance in Australia between 1995 and 2020; p=<0.001. A: Varicella vaccines were not available before 1999 [4]; B: Varicella vaccination has been incorporated in the National Immunisation Program since November 2005 [8]; X: APSU data between 1997 and 2005 were not available.

The overall incidence estimate of NVI between November 2009-November 2020 was 0.49 (95% CI 0.3–0.8) per 100,000 live births per year, which was significantly lower (p < 0.001) than the 2.1 per 100,000 live births per year reported in 2006–2009 [9] and the 5.7 per 100,000 live births per year in 1995–1997 [7] (Fig. 2). Trend analysis showed there was a 91.3% decrease in incidence of NVI during the study period compared with 1995–1997, with the number of cases declining from 44 in the first three years of surveillance (1995–1997) to 15 in the latter eleven years (late 2009–2020 (Fig. 2).

Maternal country of birth data were not available for the 1995–1997 surveillance period, however 7/17 (41%) infants with either CVS or NVI had mothers known to have been born in countries without universal VZV vaccination programs. The proportion of overseas-born mothers with VZV infected newborns increased between the 2006–2009 and 2009–2020 surveillance periods, from 36% to 41%, Odds ratio 2.4, 95% CI 0.44–3.55).

Our combined data for the 2009–2020 surveillance period showed that only two infants with NVI had received prophylaxis with Zoster immune globulin (ZIG).

## 4 Discussion

This study revealed a substantial and sustained reduction of more than 90% in the incidence of CVS and NVI between late 2009 and 2020. In our previous study (2006–2009) [9], we observed 100% and over 85% reductions in the incidence of CVS and NVI in 2008–2009 compared with 1995–1999 and 2005–2006, respectively. Our previous data showed that the fully-funded VZV vaccine administered under the Australian National Immunisation Program (NIP) for all children aged 18 months, with a catch-up program for adolescents, most likely contributed to the significant reductions in rates of CVS and NVI [9]. While the further reduction in the incidence of NVI in our current study was most likely due to the effects of ongoing VZV vaccination in the community, a further reduction in CVS incidence was less clear. Similar to our current findings, Chaves et al. [16] reported an almost 90% reduction in the incidence of varicella disease in infants aged < 12 months in the USA in the decade following the introduction of a universal vaccination program for older children. Our study demonstrates the long-term impact of universal VZV vaccination in Australia in reducing the incidence of both standard childhood varicella infection and NVI and improving the outcomes of both NVI and CVS in younger children (aged < 18 months) who are not yet eligible for VZV vaccination.

Although CVS and NVI are extremely uncommon in Australia, some high-risk cohorts require consideration. Gidding et al. [17] has demonstrated in serosurveys that up to 10% of Australian women of childbearing age (15–44 years) remain susceptible to varicella infection. To prevent CVS and NVI, women of child-bearing age without a history of varicella infection should be vaccinated [7]. An important finding from our study is that at least 41% of mothers of infected infants were born overseas in countries with no VZV vaccination program. Thus, there is opportunity to further reduce rates of maternal VZV infection and therefore CVS and NVI through public health and education programs. Furthermore, the current recommendations for health screening of Australian immigrants (including asylum seekers and refugees) for tuberculosis, hepatitis B, hepatitis C, human immunodeficiency virus (HIV) and syphilis upon arrival to Australia [18] should be amended to include screening for past varicella infection, so that at-risk individuals who have not been previously exposed can be targeted and prioritised for VZV vaccination.

Treatment with ZIG is recommended as soon as possible after exposure to protect against congenital varicella and severe maternal varicella [1], [3]. In our current study only two infants with NVI receiving ZIG, raising concern not only in vaccination coverage but potential missed opportunities to treat with ZIG.

Limitations of this study include: that only cases that came to the attention of a paediatrician were included, so cases that resulted in the termination of a pregnancy or stillbirth and seen only by and obstetrician/gynaecologist may have been missed. Thus the calculated incidence may be therefore be an underestimate of the true incidence. For five (26%) notified cases of probable neonatal varicella (one in 2010, two in 2011, one in 2012 and one in 2013) a case report form was not returned and therefore infection could not be confirmed. However, the proportion of possible cases with missing data is similar to the earlier APSU studies on varicella conducted in Australia [9]. Additionally, we have no incidence estimates between 1998 and 2005 as no APSU surveillance was conducted during this period.

## 5 Conclusions

The introduction of government-funded varicella vaccination programs have the potential to substantially reduce or eliminate the occurrence of congenital and neonatal varicella in the community. Targeted screening for varicella immunity should be considered in all young migrant, asylum seeking and refugee women entering Australia, who are at risk of varicella infection, especially those of childbearing age. Vaccination of non-immune women should then be prioritised to prevent transmission of varicella infection to their unborn and newborn children.

## Declaration of Competing Interest

The authors declare that they have no known competing financial interests or personal relationships that could have appeared to influence the work reported in this paper.

## Data Availability

Data will be made available on request.
